# Severe Blunt Liver Injury Complicated by Delayed Massive Hemobilia in a Toddler: A Case Report and Literature Review

**DOI:** 10.3389/fsurg.2022.930581

**Published:** 2022-07-08

**Authors:** Xiaoming Liu, Qianqian Sun, Wenjing Sun, Qiong Niu, Zhu Wang, Chen Liu, Tingliang Fu, Lei Geng, Xiaomei Li

**Affiliations:** ^1^ Pediatric Intensive Care Unit, Binzhou Medical University Hospital, Binzhou, China; ^2^ Department of Gastroenterology, Binzhou Medical University Hospital, Binzhou, China; ^3^ Department of Vascular and Interventional Surgery, Binzhou Medical University Hospital, Binzhou, China; ^4^ Department of Pediatric Surgery, Shanghai Children’s Hospital, Affiliated to Shanghai Jiaotong University School of Medicine, Shanghai, China; ^5^ Department of Pediatric Surgery, Binzhou Medical University Hospital, Binzhou, China

**Keywords:** blunt hepatic trauma, delayed massive hemobilia, injury prevention, toddler, case report

## Abstract

**Introduction:**

Unintentional injuries remain a leading cause of disability among children. Although most of the pediatric patients suffering blunt liver injury can be successfully treated with non-operative therapy, the diagnosis and management of delayed life-threatening hemobilia following severe blunt liver injury, especially in the pediatric population, remain a challenge for clinicians.

**Case Presentation:**

A previously healthy 2-year-old girl suffered a severe blunt liver injury related to an electric bike, which was inadvertently activated by herself. She initially received non-operative therapy and was in a stable condition in the first 2 weeks. On the 16th and 22nd postinjury days, the patient presented with life-threatening massive hemobilia, which was confirmed *via* repeat emergent gastroscopy and hepatic arterial angiography. An emergency selective transarterial embolization of the involved branch of the left hepatic artery was successfully performed. The patient recovered uneventfully, and long-term follow-up was needed owing to a mild dilatation of the left intrahepatic bile duct.

**Discussion:**

Incidental injury in children should be considered as a major public health issue and preventive measures should be taken to reduce its occurrence. Delayed massive hemobilia after severe blunt liver trauma is rare, and its accurate and timely diagnosis *via* emergency hepatic arterial angiography and selective angioembolization may allow prompt and optimal management to achieve good outcomes in the pediatric population.

## Introduction

The World Health Organization (WHO) has estimated that in the year 2004, more than tens of millions of children worldwide suffered injuries, and unintentional injuries remained a leading cause of disability among children (1). Implementation of preventive strategies will help reduce incidental injury and improve children's survival rates and health, which is vital to avoid far-reaching impacts on children's prospects and their parents’ livelihood (1–4). Today, electric bikes (e-bikes) are widely available (5, 6); however, its higher traveling speed may increase the risk of accidents and the severity of injury (6–11). Herein, we describe a rare case of a girl who suffered an e-bike-related severe blunt liver injury complicated by delayed massive hemobilia. We discuss the diagnosis and management of severe blunt liver injury associated with delayed life-threatening hemobilia in the pediatric population and review the literature in the English language from the Pubmed database.

## Case Description

**Figure 1:**
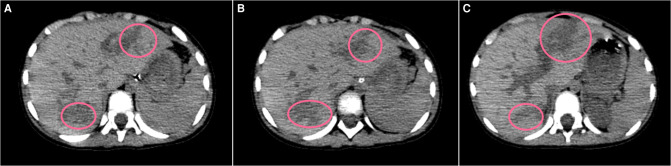
Plain CT scan (liver window **A** to **C**) showing irregular low-density lesions (pink circles) in SIVA and II, AAST Grade IV laceration and SVI, and AAST Grade III laceration.

A previously healthy girl aged 2 years weighing 15 kg was brought to our pediatric emergency department and admitted 5 h after her chest and upper abdomen were squeezed by an e-bike with high traveling speed in her family yard. The e-bike was inadvertently activated by herself, and it suddenly moved forward and pushed her chest and abdomen into a brick wall. On admission, she was conscious but looked pale. There were signs of hemorrhagic shock, blood pressure of 103/49 mmHg, heart rate of 167 beats/min, and respiratory rate of 42 breaths/min. Bruises were observed on her right lateral chest wall. Her abdomen revealed slight tenderness and was distended. The laboratory tests showed a red blood cell (RBC) count of 3.29 ×  10^12^/L, hematocrit of 25%, and a hemoglobin (Hb) level of 90 g/L. Liver function test revealed an elevated liver enzyme, aspartate aminotransferase (AST) of 2,898 IU/L, and alanine aminotransferase (ALT) of 2,247 IU/L. Chest and abdominal plain radiograph was unremarkable. Chest and abdominal plain computed tomography (CT) revealed bilateral traumatic wet lungs and irregular lacerations in segments VI, IVA, and II, graded as III and IV, respectively, by the American Association for the Surgery of Trauma's Organ Injury Scaling (AAST-OIS) (12) (Figure 1). After admission, the Hb level declined to 71 g/L. Point-by-care ultrasonography demonstrated that the involved area of the left hepatic lobe was 5.5 cm × 4.8 cm × 1.5 cm and that of the right lobe was 4.8 cm × 4.4 cm × 4.9 cm with hemoperitoneum. Initial fluid resuscitation, blood transfusion of two units of concentrated RBCs, and hemostatics were given immediately. The patient received non-invasive ventilation with high volume oxygen inhalation and absolute bed rest. The Hb level was within the normal range. The patient received non-operative management and recovered uneventfully. On the 11th postinjury day, she was transferred to a conventional ward.

**Figure 2:**
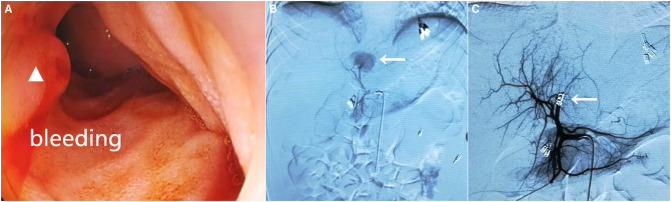
Gastroduodenoscopy revealing active bleeding with fresh blood from the duodenal papilla (white triangle) (**A**). Hepatic arteriography showed contrast extravasation from the branch of the left hepatic artery (white arrow) (**B**) and selective transcatheter arterial embolization was successfully implemented (**C**, white arrow).

On the 16th postinjury day, the patient suddenly presented abdominal discomfort and hematemesis. Then, massive bloody stool occurred with hemorrhagic shock. The Hb level declined to 52 g/L with hyperbilirubinemia (total bilirubin 99.8 mmol/L, direct bilirubin 82.3 mmol/L). Urgent fluid resuscitation, blood transfusion, systemic hemostatics, and oxygen inhalation were given. Emergent gastroscopy revealed no obvious bleeding from the duodenal papilla, and erosive gastroduodenitis was suspected. The source of gastrointestinal bleeding was unclear, but the bleeding ceased spontaneously. The Hb level was 103 g/L following multiple blood transfusions of 6 units of concentrated RBCs. On the 22nd postinjury day, massive bloody stool reoccurred. The Hb level dramatically declined to 51 g/L. Emergent re-gastroscopy showed active bleeding from the duodenal papilla (Figure 2A). The diagnosis of traumatic massive hemobilia was confirmed. After receiving fluid resuscitation and blood transfusions of 5 units of concentrated RBCs, the patient underwent an emergency hepatic arteriography. A pseudoaneurysm from a branch of the left hepatic artery was confirmed and contrast extravasation under the diaphragm was also observed. The involved arterial branch was selectively embolized using gelatin sponge particles, spring coil, and free coil. Post-embolization angiography showed a complete occlusion of the pseudoaneurysm and no contrast medium extravasation (Figures 2B,C). The patient recovered uneventfully and was discharged on the 5th post-embolization day.

After a one-year follow-up, regular ultrasonography and color Doppler ultrasound showed that a heterogeneous echogenicity in the parenchyma of the left hepatic lobe and the inner diameter of the portal vein was normal. Echogenicity of blood clots in the gallbladder lumen gradually disappeared at 2 months post-embolization. A mild dilatation of the left intrahepatic bile duct was found at the 6th week after trauma, and long-term follow-up was needed.

The Pubmed database revealed that there were about 35 pediatric patients who suffered blunt liver injury complicated by hepatic artery pseudoaneurysm and/or delayed life-threatening hemobilia (12–41). The clinical characteristics of these patients, including those of the present one, are summarized in Table 1.

**Table 1:** Summary of the clinical characteristics (*n* = 36).

Age (years)	
2–7	12
8–12	13
13–18	8
Unknown	3
Gender	
Male	17
Female	9
Unknown	10
Mechanism of injury	
Bicycle handlebar collision	10
Fall	6
Car or electric bike accident	5
Other (struck by a piece, kicked by a horse, gunshot injury, punched by a classmate)	6
Unknown	9
Location of the pseudoaneurysm	
Right lobe of the liver	30
Left lobe of the liver	6
Time of hemobilia or pseudoaneurysm confirmed from trauma	
1 day–2 weeks	26
>2 weeks	7
Unknown	3
Clinical manifestation	
Abdominal pain (tenderness)	9 (12)
Jaundice	7
Hematemesis and malena	31
Hypotension	10
Investigation	6
Color Doppler ultrasound (a round echo-poor lesion with turbulent blood flow)	6 (2)
An echogenic blood clot in the gallbladder by ultrasonography	8
Computerized tomographic angiography (positive)	19 (18)
Hepatic artery angiography (showing pseudoaneurysm, contrast extravasation)	15 (14, 6)
Gastroscopy (showing bleeding from the Vater's ampulla)	9 (3)
Multiple injuries	
Diaphragm rupture	2
Kidney injury	2
Spleen injury	2
Fracture of rib, clavicle, pelvis	3
Other (pancreas, perforated duodenum, wet lung)	3
Management	
Non-operative therapy (observed by angiography or cholangiography)	4
Selective arterial embolization (re-embolization)	24 (2)
Surgically involved hepatic artery or its branch ligation (biliary duct repair)	7 (1)
Massive liver resection or redo operation	3
Outcome	
Alive	35
Follow-up	
Doing well	34
Decreased intellectual capacity and motor ataxia	1
Complications	
Mild biliary duct dilatation	1
Subphrenic and pelvic abscesses	1

## Discussion

The WHO reports that many children suffering nonfatal injuries are left with some form of disability, even with lifelong consequences (1–3). E-bike, as one of the new personal mobility devices, is being widely used for the last two decades (42). This convenient mode of transportation leads to a high ratio of e-bike-related casualties (9, 10, 43–45). If e-bike riders leave the key *in situ* after riding, toddlers may easily activate it inadvertently owing to their high curiosity and imitative ability. This can lead to their being exposed to a high risk of severe injuries. In the present case series, bicycle riding and fall-related high-grade liver injury occurred in nearly half of the patients (16/35), as shown in Table 1.

The patient in this study who suffered an e-bike-related severe blunt liver injury, accompanied by a wet lung, was initially treated non-operatively during the first 2 weeks after trauma, as described in previous reports (15, 16, 46). However, at the 3rd week after trauma, the patient suddenly presented with massive upper gastrointestinal bleeding with hemorrhagic shock, which required a high volume of blood transfusion. The diagnosis and positioning of the ruptured hepatic artery pseudoaneurysm were confirmed *via* repeat urgent gastroscopy and emergency hepatic angiography in the second episode of bleeding. Fortunately, a selective transcatheter angiographic embolization was successfully performed with good outcome.

Traumatic hepatic artery pseudoaneurysm, as a complication, may present as a clinically silent hemorrhage or as a sudden massive hemorrhage. Prompt recognition and a multidisciplinary approach with an experienced team are needed to manage this rare but life-threatening condition, especially in the pediatric population (17, 18, 32, 34, 36–39, 47, 48). Patients with AAST-OIS high-grade blunt hepatic injury seem to be at a higher risk of developing pseudoaneurysm based on a retrospective study. Pseudoaneurysm is usually identified within 2 weeks after liver trauma, as shown in Table 1. Therefore, routine surveillance may be indicated and should benefit patients because some of them present with sudden massive hemobilia after discharge (13, 48–52). Patients with traumatic hemobilia may present with a triad of the upper abdominal pain, hemobilia, and obstructive jaundice (19, 53, 54). However, there are a variety of presentations according to the source and degree of hemorrhage (36, 51, 55). The early diagnosis of hemobilia mainly depends on a high index of suspicion for those who suffered blunt liver injury followed by upper gastrointestinal bleeding and biliary symptoms (35, 49). Occasionally, pseudoaneurysm may rupture into the peritoneal cavity (15, 33). However, in the present case, hepatic angiographic imaging revealed a pseudoaneurysm from one branch of the left hepatic artery, and contrast media extravasation was seen under the diaphragm, suggesting that there were communications between the involved artery to both bile ducts and the abdominal cavity. This angiographic finding is extremely rare (42, 55) and should be managed accordingly.

A precise diagnosis is mainly made *via* contrast-enhanced CT and/or angiography (34, 36, 55). As far as the role of gastroscopy in the diagnosis of hemobilia is concerned, blood clots in the duodenum may often be revealed (25, 33). There are only two of the nine cases revealing bleeding from Vater's ampulla, as shown in Table 1. Repeat gastroscopy may be helpful to make the diagnosis of hemobilia. The selective use of point-of-care ultrasound imaging in children with high-grade liver injury may allow to identify hepatic artery pseudoaneurysms, which might minimize the risk of life-threatening hemorrhage (19). “Yin-yang” sign is the classic manifestation in color Doppler ultrasound for artery pseudoaneurysm (56).

**Figure 3:**
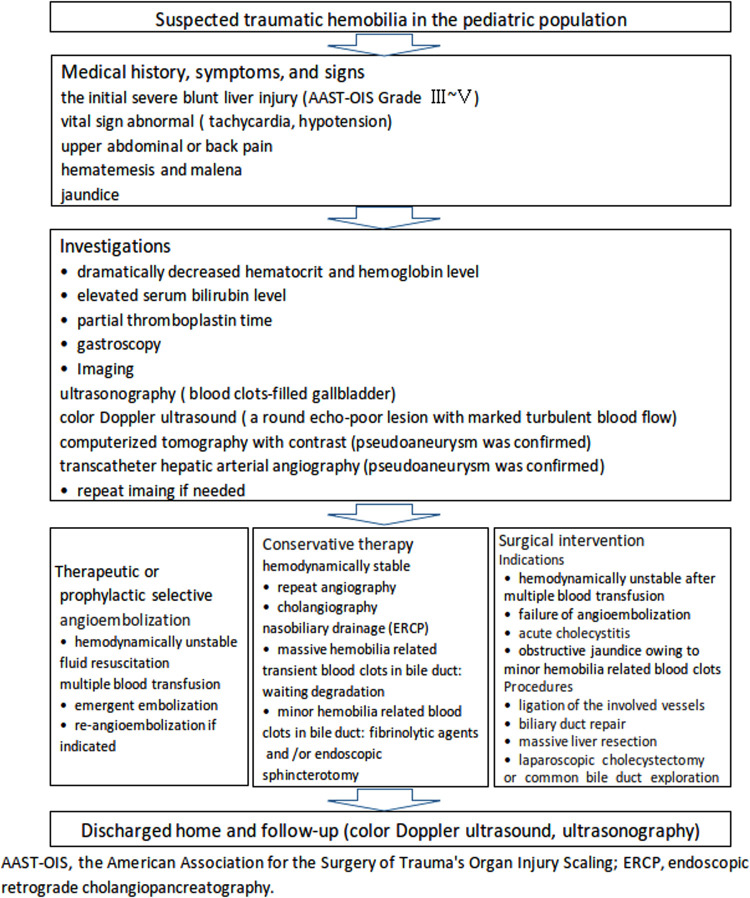
A recommended flowchart for the diagnosis and management of traumatic hemobilia in the pediatric population based on the literature and our preliminary experience.

Management of traumatic hemobilia is aimed at stopping bleeding and relieving biliary obstruction (36). Conservative management includes fluid resuscitation, blood transfusion, correction of coagulopathy, and adequate drainage (36). Endoscopic nasobiliary drainage without sphincterotomy is an optimal method to treat traumatic hepatobiliary injuries in patients with stable hemodynamics (57). Hemobilia may stop because of blood clot formation after the diversion of bile with nasobiliary drainage (57). Selective transcatheter arterial embolization as a useful therapeutic method for traumatic hepatic pseudoaneurysm and/or its rupture into the biliary duct remains the first-line choice for the majority of cases (17, 31, 35, 36, 39, 40, 50, 55, 58), with 26/35 (74.28%) of patients undergoing successful angiographic embolization, as shown in Table 1. Surgical procedures still play an important role in the management of patients with unstable hemodynamics or failure following selective angiographic embolization (22, 27, 36, 50, 59). A flowchart (Figure 3) was recommended to provide a suitable approach to deal with this rare but life-threatening condition in accordance with the literature (15, 17, 33, 36, 46, 50, 52, 54–64) and our preliminary experience.

Short-term complications, such as cholecystitis, gallbladder necrosis, liver ischemia, abscess, rebleeding, and febrility should be properly treated (19, 36, 52, 54, 59). Long-term follow-up is needed as complications may occur after liver trauma and angioembolization (36, 39).

In conclusion, massive upper gastrointestinal bleeding in patients with a history of severe blunt liver trauma should be highly suspicious of hemobilia. The diagnosis can be made *via* CT angiography and hepatic artery angiography (16, 17, 36, 41). Based on the age-specific characteristics of children, a multidisciplinary team, especially experienced interventional radiologists, is vital for the diagnosis and management of this rare but life-threatening condition to achieve a good outcome (18, 20, 37). Moreover, the management of e-bike-related injuries should not be underestimated in the pediatric population (5, 8, 9). Preventive measures, such as the promotion of safety devices and safety education, should be taken to reduce unintentional injuries among children.

## Data Availability

The raw data supporting the conclusions of this article will be made available by the authors, without undue reservation.
